# Establishment and application of a rapid molecular diagnostic platform for the isothermal visual amplification of group B Streptococcus based on recombinase polymerase

**DOI:** 10.3389/fcimb.2024.1281827

**Published:** 2024-02-23

**Authors:** Meilin Liu, Huan Wang, Chu Chu, Fanli Min, Lizhou Sun, Teng Zhang, Qian Meng

**Affiliations:** ^1^ Obstetrical Department, Lianyungang Maternal and Child Health Hospital, Lianyungang, Jiangsu, China; ^2^ Obstetrical Department, The First Affiliated Hospital of Nanjing Medical University, Nanjing, Jiangsu, China

**Keywords:** group B streptococcus, cfb, recombinase polymerase, isothermal amplification, molecular diagnostic methods

## Abstract

With growing concerns about Group B streptococcal (GBS) infections and their adverse effects on perinatal pregnancies, including infection, premature delivery, neonatal septicemia, and meningitis, it is urgent to promote GBS screening at all pregnancy stages. The purpose of this study is to establish a device-independent, fast, sensitive, and visual GBS detection method. Taking advantage of the characteristics of the recombinase polymerase isothermal amplification (RPA), the activity of the nfo nuclease cleavage base analog (tetrahydrofuran, THF) site, and the advantages of visual reading of the lateral flow chromatography strip (LFS), a GBS detection method was developed. This method focused on the conservative region of the Christie–Atkins–Munch–Petersen factor encoded by the cfb gene, a virulence gene specific to GBS. Two forward primers, two biotin-labeled reverse primers, and one fluorescein isothiocyanate (FITC)-labeled and C3spacer-blocked probe were designed. The study involved optimizing the primer pair and probe combination, determining the optimal reaction temperature and time, evaluating specificity, analyzing detection limits, and testing the method on 87 vaginal swabs from perinatal pregnant women. The results showed that the visual detection method of GBS-RPA-LFS, using the cfb-F1/R2/P1 primer probe, could detect GBS within 15 min at the temperature ranging from 39°C to 42°C. Furthermore, the method specifically amplified only GBS, without cross-reacting with pathogens like *Lactobacillus iners*, *Lactobacillus crispatus*, *Candida albicans*, *Listeria monocytogenes*, *Yersinia enterocolitica*, *Klebsiella Pneumoniae*, *Enterobacter cloacae*, *Citrobacter freundii*, *Vibrio alginolyticus*, *Vibrio parahaemolyticus*, *Salmonella typhimurium*, *Staphylococcus aureus*, *Pseudomonas aeruginosa*, or *Trichomonas vaginalis*. It could detect a minimum of 100 copies per reaction. In clinical 98 samples of vaginal swabs from pregnant women, the agreement rate between the GBS-RPA-LFS method and TaqMan real-time fluorescence quantification method was 95.92%. In conclusion, this study successfully established a combined RPA and LFS GBS *in situ* detection platform, with short reaction time, high sensitivity, high specificity, portability, and device independence, providing a feasible strategy for clinical GBS screening.

## Introduction

Group B Streptococcus (GBS), also known as *Streptococcus agalactiae*, is a commonly encountered opportunistic pathogen. This aerobic, β-hemolytic, gram-positive coccus typically colonizes the lower digestive tract and urogenital tract (Raabe and Shane, 2019). It is an important human pathogen, posing a serious threat particularly to pregnant women and newborns (Yuan et al., 2021). The carrier rate among healthy individuals is up to 15%–35% (Otaguiri et al., 2013), and carriers are often asymptomatic (Brigtsen et al., 2022). GBS is an important pathogen of severe infection such as neonatal pneumonia, septicemia, and purulent meningitis and can lead to poor pregnancy outcomes like abortion in women with low immunity (Okike et al., 2014; Korir et al., 2017; Chen et al., 2019). For neonates, GBS infections occurring before 28 weeks of gestation are associated with higher mortality, and premature infants born before 34 weeks are at increased risk for complications, disabilities, and mortality (Le Gallou et al., 2023). As early as the 1990s, the US Centers for Disease Control and Prevention (CDC) has carried out antibiotic interventions for pregnant women testing GBS positive at 35–37 weeks of gestation to improve pregnancy outcomes and reduce neonatal infections and complications (Cotten, 2015). However, GBS is not currently included in prenatal screening programs in China, and awareness among pregnant and postpartum women in China needs further improvement.

Common GBS detection methods include bacterial culture, immunological testing, and molecular diagnostic methods (Chalifour et al., 1992; Carrillo-Ávila et al., 2018; Shin and Pride, 2019; Tiruvayipati et al., 2021). Bacterial culture is considered the gold standard for GBS detection. This method involves collecting vaginal and rectal swabs from pregnant women at 35–37 weeks of gestation and culturing them in a broth medium. The final identification of GBS is based on colony morphology, hemolytic characteristics, bacterial morphology observation, and biochemical reactions (Nickmans et al., 2012). However, bacterial culture has several drawbacks, including a lengthy diagnostic time of 48 h–72 h, complex result interpretations, and cumbersome operation steps (Carrillo-Ávila et al., 2018). Immunological assays, which are based on antigen–antibody responses, have a shorter detection cycle but tend to have slightly lower sensitivity and specificity (Hill et al., 1975; Chalifour et al., 1992; Ghaddar et al., 2014). The presence of numerous serotypes in GBS can lead to missed detections, limiting the clinical application of these assays (Raff et al., 1988). Molecular diagnostic methods, such as nucleic acid probe detection, offer high sensitivity but are costly. Polymerase chain reaction (PCR)-based methods provide strong sensitivity and high specificity but require expensive and precise equipment, which limits their widespread use (Shin and Pride, 2019). In addition, proteomic-based analysis methods, although accurate and fast, also rely on expensive mass spectrometers.

Considering that GBS poses a serious threat to pregnant women and newborns, it is particularly important to establish universally applicable, cost-effective GBS testing methods. These methods must be accurate, sensitive, simple to operate, and easy to read while also being feasible in laboratories with limited resources and in remote areas. Comprehensive consideration of the above factors is essential to meeting market demand for a GBS testing method. We propose developing a new rapid GBS detection method using the recombinase polymerase amplification (RPA) technique. RPA, a revolutionary nucleic acid amplification technology, employs core enzyme components T4 UvsY, T4 UvsX, gp32, and Bsu to efficiently complete DNA amplification at a constant low temperature (approximately 37°C–42°C). This process requires no complex equipment and special experimental conditions, enabling equipment-independent GBS detection (Piepenburg et al., 2006). Although the basic RPA technique has been used for GBS detection, the interpretation of its results is still limited to well-equipped laboratories (Yu et al., 2023).

**Figure 1:**
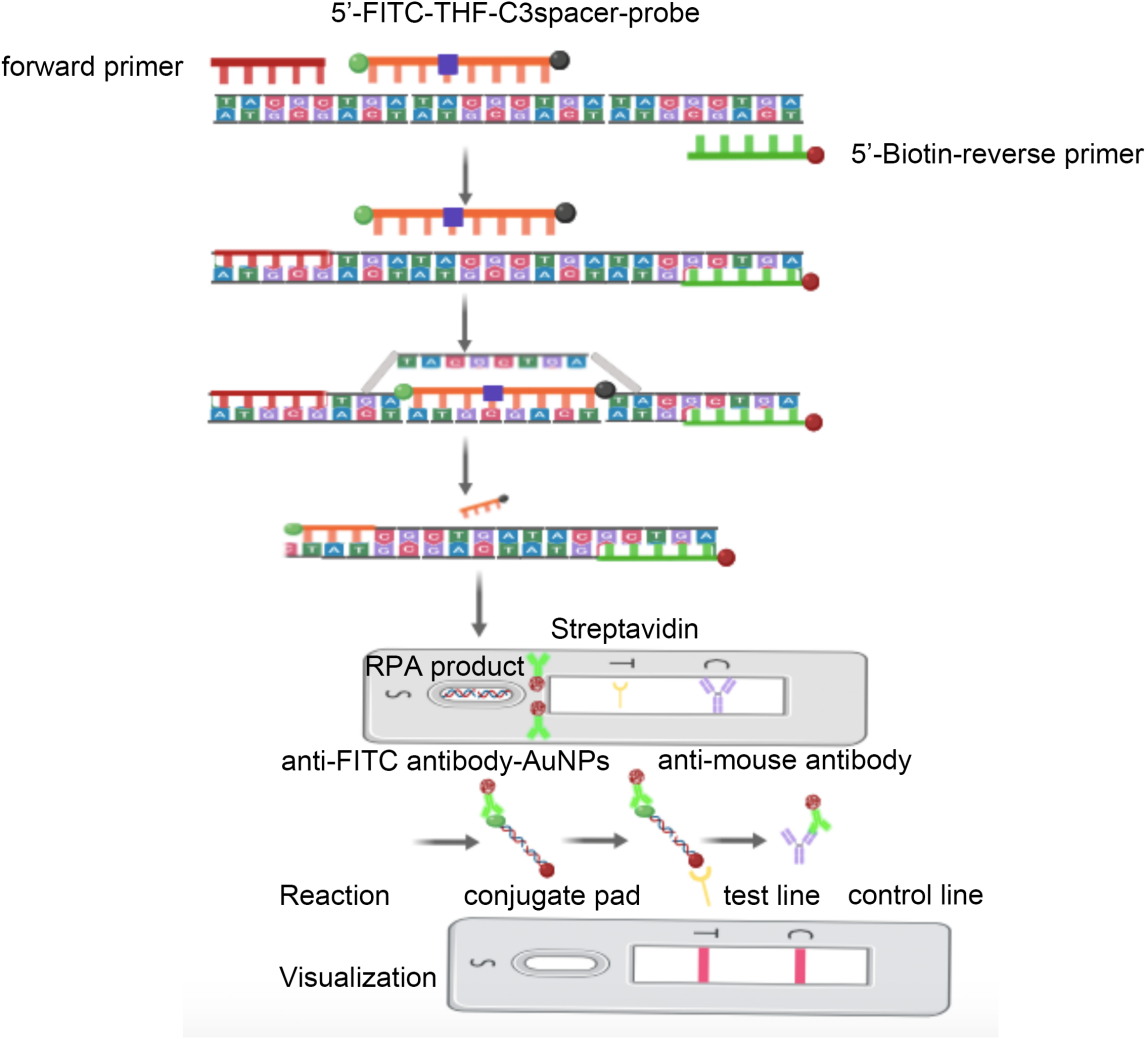
Schematic of RPA amplification and LFS visualization.

By introducing nfo enzymes and fluorescent labeling groups during RPA amplification, the accumulated DNA products can be visualized with lateral flow chromatography strips (LFS) (Hu et al., 2019) and some portable blue light and UV detectors (Huang et al., 2023). However, detection based on fluorescent probes may yield false positive background signals, leading us to use LFS to establish the detection method in this study. In order to improve reaction specificity and make the visual reading of the results possible, we propose adding a fluorescein isothiocyanate (FITC) antigen-labeled probe to the RPA system and introducing a base analog (tetrahydrofuran, THF) site. To prevent it from unconditionally replacing the forward primer for amplification, we applied a C3Spacer blocking at its 3′ end. For accumulating RPA products on the LFS detection line, we labeled the 5′ end of the reverse primer with biotin, which could bind to streptavidin coated on the LFS detection line. When LFS is inserted into the RPA product, all FITC containing DNA undergo antigen–antibody reactions with anti-FITC antibodies bound to gold nanoparticles (AuNPs). When the FITC containing a DNA product flows to the detection line coated with streptavidin, recognized by biotin-specific binding, only positive RPA products are intercepted, showing the accumulation and coloration of AuNPs. When all FITC containing DNA continues to flow to the control line coated with anti-FITC primary antibody, the AuNPs will still aggregate for color rendering (**Figure 1**). By combining RPA technology with nfo enzymes and GBS-specific virulence genes (Mudzana et al., 2021; Awwad et al., 2022), like the Christie–Atkins–Munch–Petersen (CAMP) factor encoded by a cfb virulence gene (Koide et al., 2022; Shi et al., 2022), we plan to design RPA amplification primers and LFS detection probes specifically targeting GBS for rapid and accurate detection.

This study aims to establish an on-site GBS-RPA-LFS visualization detection platform based on the cfb gene. By utilizing the isothermal amplification characteristics of recombinase polymerase, the cleavage of THF sites by the nfo enzyme, and the advantages of LFS’s visualized reading, GBS amplification will be completed under a portable and low-cost constant temperature heater. The detection results for GBS will be read through direct visual inspection. The platform will undergo analysis for exclusivity against other interfering pathogens, as well as a sensitivity analysis for detection. Additionally, we will conduct a consistency evaluation of pregnant women’s recto-vaginal swabs using the TaqMan real-time fluorescence quantification method to establish a reliable and valuable GBS screening method.

## Materials and methods

### Ethics statement

This study was approved by the Ethics Committee of Lianyungang Maternal and Child Health Care Hospital (permission number: XM2023013). The urine and recto-vaginal swab samples were collected by the Gynecology of Lianyungang Maternal and Child Health Care Hospital from May to July 2023. Each pregnant woman participating in the study provided consent and signed an informed consent form.

### Sequence alignment of the cfb gene

The nucleotide sequences of the cfb genes from different strains of *Streptococcus agalactiae* published by NCBI, as published by NCBI, were downloaded for analysis. These strains include ATCC 27956 (GenBank: MK134700.1) and strain GRS2061 (GenBank: JQ289586.1), strain GRS2058 (GenBank: JQ289585.1), strain GRS2059 (GenBank: JQ289584.1), strain GRS2066 (GenBank: JQ289583.1), and strain GRS0567 (GenBank: JQ289582.1). The DNAMAN software was used for homology analysis of conserved regions.

### Design of primers and probes based on the cfb gene

According to the nucleotide sequence of the cfb gene of *S. agalactiae* (strain ATCC 27956) published by NCBI (GenBank: MK134700.1), two specific forward primers and two specific reverse primers of RPA were designed within its conserved region using the “Pick Primers” function of NCBI. The Primer Premier 3 software was used to design specific probes that match between the forward and reverse primers. All primers and probes were verified for specificity by NCBI’s “Primer BLAST” function and synthesized by General Biotech (Anhui, China), as detailed in **Table 1**. Specifically, we need to label the 5′ end of the reverse primer with biotin, label the 5′ end of the probe with FITC, insert a THF site in the middle of the probe, and block the 3′ end of the probe with C3spacer (Dong et al., 2020).

**Table 1:** Primer and probe information used in this study.

Primers	Sequence information (5′–3′)
cfb-F1	ATGAACGTTAAACATATGATGTATC
cfb-F2	TATCTGGAACTCTAGTGGCTGGTGCA
cfb-R1	Biotin-GTCTCAGGGTTGGCACGCAATGAAG
cfb-R2	Biotin-TAAACTGTCTCAGGGTTGGCACGCA
cfb-P1	FITC-TCAAGCCCAGCAAATGGCTCAAAAGC(THF)TTGATCAAGATAGC AT-C3spacer

### Preparation of genomic DNA of bacterial and parasite strains

*S. agalactiae*, isolated in our hospital, was confirmed by PCR to have 100% homology with the CAMP factor of *S. agalactiae* strain ATCC 27956. This strain will be used to establish the GBS detection method in subsequent experiments. Details of other clinically isolated pathogens are provided in **Table 2**. All strains were stored at a concentration of 10^7^ CFU/mL and *Trichomonas vaginalis* at a concentration of 10^3^ parasites/mL. After centrifugation at 12,000 × g for 1 min, the bacterial suspension were resuspended in an equal volume of TE buffer and heated at 100°C for 10 min to fully release genomic DNA. The genomic DNA of *T. vaginalis* was extracted by TIANamp Genomic DNA Kit and used in follow-up experiments.

**Table 2:** Bacterial and parasite strains information used in this study.

Pathogen name	Source	Concentration (in TE buffer)
*Streptococcus agalactiae*	ATCC 27956 (100% homology of cfb gene)	10^7^ CFU/mL
	Isolate FYR0615	10^7^ CFU/mL
	Isolate WML0617	10^7^ CFU/mL
	Isolate WCP0617	10^7^ CFU/mL
	Isolate JZK0613	10^7^ CFU/mL
	Isolate XSY0613	10^7^ CFU/mL
	Isolate MH0610	10^7^ CFU/mL
	Isolate SLL0610	10^7^ CFU/mL
	Isolate ZTT0620	10^7^ CFU/mL
*Lactobacillus iners*	B308112 (Mingzhoubio)	10^7^ CFU/mL
*Lactobacillus crispatus*	BMZ133559 (Mingzhoubio)	10^7^ CFU/mL
*Candida albicans*	ATCC 10231	10^7^ CFU/mL
*Listeria monocytogenes*	ATCC 19115	10^7^ CFU/mL
*Yersinia enterocolitica*	ATCC 23715	10^7^ CFU/mL
*Klebsiella pneumoniae*	NCTC 8172	10^7^ CFU/mL
*Enterobacter cloacae*	BMZ134831	10^7^ CFU/mL
*Citrobacter freundii*	BMZ122577	10^7^ CFU/mL
*Vibrio alginolyticus*	ATCC 17749	10^7^ CFU/mL
*Vibrio parahaemolyticus*	ATCC 17802	10^7^ CFU/mL
*Salmonella typhimurium*	ATCC 14028	10^7^ CFU/mL
*Staphylococcus aureus*	ATCC 6538	10^7^ CFU/mL
*Pseudomonas aeruginosa*	ATCC 27853	10^7^ CFU/mL
*Trichomonas vaginalis*	Isolated strain	10^3^ parasites/mL

### RPA amplification and LFS visual detection

The cfb gene of GBS was amplified by RPA according to the instruction of the TwistAmp DNA Amplification nfo kit. The 50-µL reaction system contained 29.5 µL of rehydration buffer, 2.1 µL of 10 µM forward and reverse primers, 0.6 µL of 10 µM probe, 1 µL of template (bacterial or parasite strains), 12.2 µL of nuclease-free water, the RPA core enzyme reaction pellet, and 2.5 µL of 280 mM magnesium acetate. The reaction was conducted at 37°C for 30 min. In particular, magnesium acetate should to be added to the top of the reaction tube before being incorporated into the RPA reaction system to initiate the amplification. The nucleic acid amplification products were detected according to the instructions of the LFS. A total of 50 µL of the RPA amplification product was added to the sample loading well. The sample was allowed to flow through the conjugate pad, the detection line, and the control line, in that order, until it stabilized. The results were recorded by reading the color development of the gold nanoparticles within 5 min.

### Screening for the optimal RPA reaction temperature

RPA reaction tubes with the same composition were individually placed in metal heaters at temperatures of 16°C, 25°C, 30°C, 37°C, 39°C, 42°C, 50°C, 60°C and heated for 30 min. The optimal RPA reaction temperature was determined through LFS analysis.

### Screening for the optimal RPA reaction time

The optimal RPA reaction time was determined through LFS analysis. Reaction tubes with the same composition were heated at the optimal reaction temperature for 10, 15, 20, 25, 30, 35, 40, and 45 minutes, respectively.

### Specificity evaluation

To evaluate the specificity of the RPA-LFS detection system for the GBS cfb gene, heat-cleaved genomic DNA samples from *L. iners*, *L. crispatus*, *C. albicans*, *L. monocytogenes*, *Y. enterocolitica*, *K. Pneumoniae*, *E. cloacae*, *C. freundii*, *V. alginolyticus*, *V. parahaemolyticus*, *S. typhimurium*, *S. aureus*, *P. aeruginosa*, and *T. vaginalis* were used as templates.

### Preparation of artificially contaminated urine samples and genomic DNA extraction

Fresh urine samples were centrifuged at 12,000 × g for 1 min, and the supernatant was collected and supplemented with EDTA to achieve a final concentration of 5 mM (Guo et al., 2021). The mixture was used to resuspend the *S. agalactiae* bacterial solution. Genomic DNA was released using the thermal lysis method, which involves boiling in a 100°C water bath for 10 min. Purified genomic DNA was prepared using a genomic DNA extraction kit.

### Determination of detection sensitivity

*S. agalactiae* crude bacterial solutions, crude-contaminated urine samples, and purified genomic DNA from contaminated urine samples were serially diluted in a 10-fold gradient with TE buffer to achieve final concentrations ranging from 10^5^ copies/μL–10^1^ copies/μL. These dilutions were then used as templates in the RPA-LFS under the optimal reaction conditions determined from screening to assess the detection sensitivity of this method.

### Detection of the cfb gene using TaqMan probe-based real-time fluorescence quantitative PCR

Specific gene primers were synthesized based on the cfb gene with the following sequences: TaqMan qPCR-F: GAAACATTGATTGCCCAGC, TaqMan qPCR-R: AGGAAGATTTATCGCACCTG. The probe sequence is as follows: TaqMan qPCR-P: FAM-CCATTTGATAGACGTTCGTGAAGAG-BHQ-1 (Carrillo-Ávila et al., 2018). The expected size of the amplicon is 99 bp. According to the AceQ U+ Probe Master Mix kit instructions, a 20-µL reaction system was prepared containing 10 µL of 2 × AceQ U+ Probe Master Mix, 0.4 µL of 10 µM TaqMan qPCR-F/R, 0.2 µL of 10 µM TaqMan qPCR-P, 0.4 µL of 50 × ROX Reference Dye 2, and 9 µL of ddH_2_O. The reaction program was set as follows: a decontamination reaction with 37°C heat treatment for 2 min, pre-denaturation at 95°C for 5 min, followed by 45 cycles of denaturation at 95°C for 10 s and annealing/extension at 60°C for 30 s. The whole reaction was conducted on an ABI 7500 fluorescence quantitative PCR instrument.

### Comparison of RPA-LFS and TaqMan qPCR applications in clinical vaginal swab samples

A total of 98 recto-vaginal swab samples from pregnant women, including the lower 1/3 of the vaginal segment and the anal sphincter area 2 cm to 5 cm inside, were clinically collected and vigorously mixed with TE buffer. After centrifugation at 3,000 × g at 4°C for 5 min, the supernatant was collected, and genomic DNA was extracted using the heat lysis method, which involved boiling in a 100°C water bath for 10 min. The genomic DNA was stored at −20°C, and all samples were tested within 1 week of collection using both the RPA-LFS method and TaqMan probe-based qPCR detection.

## Results

### Screening of cfb gene-specific RPA primers and probes for the GBS target

**Figure 2:**
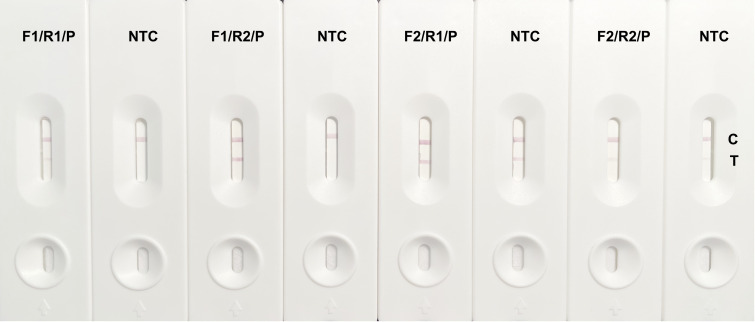
Results of cfb gene-specific RPA primers and probe combination screening. The cfb gene of GBS was amplified by RPA and detected by LFS visualization with combinations of cfb-F1/R1/P, cfb-F1/R2/P, cfb-F2/R1/P, and cfb-F2/R2/P. Each combination is identified above its corresponding LFS, which is marked by a control line (C) and a detection line (T). The experimental group that used nuclease-free water instead of a template served as the negative control (NTC).

A homology analysis was performed on the cfb gene of six *S. agalactiae* isolates, using the nucleotide sequence from strain ATCC 27956 as a reference. The homology of the cfb gene from strain ATCC 27956 to isolates GRS2061, GRS2058, GRS2059, GRS2066, GRS0567, and GRS2063 was found to be 98.96%, 99.87%, 98.44%, 98.57%, 99.48%, and 98.57%, respectively, indicating significant conservation. Consequently, two specific upstream primers, cfb-F1 and cfb-F2, two specific downstream primers, cfb-R1 and cfb-R2, and one probe, cfb-P, were designed between the upstream and downstream primers. Pairwise combinations of these primers and probes were tested, and primer controls with nuclease-free water were used for each combination to analyze the sensitivity and specificity of the primer–probe pairs. As shown in **Figure 2**, the experimental groups of all four combinations showed aggregation of gold nanoparticles at both the detection and control lines. The amplification sensitivity of the cfb-F1/R2/P combination was comparable with that of cfb-F2/R1/P and higher than that of cfb-F2/R2/P, whereas cfb-F1/R1/P exhibited the lowest sensitivity. However, aggregation of gold nanoparticles was observed at the detection line in the negative control group of cfb-F2/R1/P and cfb-F2/R2/P, even without a template, with the non-specific band in cfb-F2/R1/P being particularly noticeable. Therefore, the combination cfb-F1/R2/P was selected for subsequent experiments.

### Screening of the optimum reaction temperature and time for GBS detection using RPA-LFS

**Figure 3:**
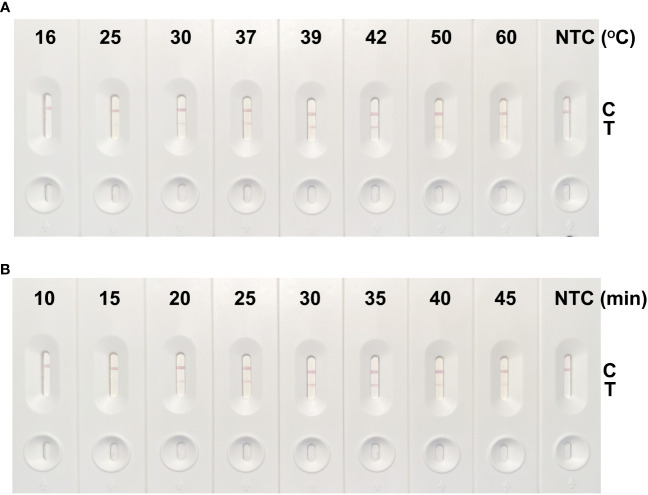
Screening results of the optimal reaction temperature and time of the GBS detection method using RPA-LFS. **(A)** The cfb-F2/R1/P primer and probe combination was used for reactions at 16°C, 25°C, 30°C, 37°C, 39°C, 42°C, 50°C, and 60°C, each for 30 min. The optimal reaction temperature was screened by LFS detection. **(B)** The reaction times were 10 min, 15 min, 20 min, 25 min, 30 min, 35 min, 40 min, and 45 min at 39°C, and the optimum reaction time was screened by LFS detection.

We set reaction temperature parameters commonly used in the laboratory, and **Figure 3A** shows the results of RPA amplification of the cfb gene at different temperatures. From 16°C to 60°C, no cfb product was accumulated at 16°C and 25°C. From 30°C, a weak cfb product band appeared as a result of RPA amplification. With the increase of reaction temperature, the enzyme activity of RPA increased, its amplification ability increased, and the cfb gene product accumulated. At 39°C, the product levels stabilized and continued to 42°C. However, the amplification performance of RPA for the cfb gene decreased at 50°C. Therefore, we identified the temperature range of 39°C–42°C as the optimal amplification temperature for this study.

**Figure 3B** shows the results of the amplification of the cfb gene by RPA at 39°C for different reaction times. A faint cfb product band was observed starting at 15 min, across a time range of 10 min to 45 min. With the extension of reaction time, cfb products were accumulated, and there was an accumulation of cfb products, with the production becoming stable from 30 min onward. No obvious increase in product accumulation was observed between 30 min and 45 min. Therefore, we determined 30 min to be the optimal amplification time for this method.

### Evaluation the specificity of GBS detection using RPA-LFS in different bacteria and parasite strains

**Figure 4:**
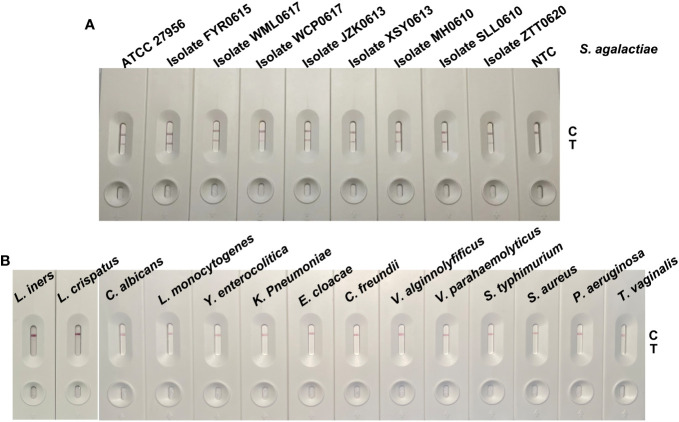
Interspecific detection results of the RPA-LFS-based GBS detection method. Thermally cleaved genomic DNA of **(A)** Streptococcus agalactiae (ATCC 27956 and 8 isolates) and **(B)** *L. iners*, *L. crispatus*, *C. albicans*, *L. monocytogenes*, *Y. enterocolitica*, *K. Pneumoniae*, *E. cloacae*, *C. freundii*, *V. alginolyticus*, *V. parahaemolyticus*, *S. typhimurium*, *S. aureus*, *P. aeruginosa*, *T. vaginalis* were used as templates. A no template control (NTC) was also established. The RPA-LFS-based GBS detection method was conducted under 39°C for 30 min, and the results were detected by LFS.

To evaluate the detection specificity of the selected cfb genes in different pathogens, we tested the cfb genes in *S. agalactiae* (ATCC 27956 and 8 isolates), *L. iners*, *L. crispatus*, *C. albicans*, *L. monocytogenes*, *Y. enterocolitica*, *K. Pneumoniae*, *E. cloacae*, *C. freundii*, *V. alginolyticus*, *V. parahaemolyticus*, *S. typhimurium*, *S. aureus*, *P. aeruginosa*, and *T. vaginalis*. As shown in **Figure 4**, only *S. agalactiae* (ATCC 27956 and eight isolates) could be detected by the established detection system, and no accumulation of cfb gene amplification products was found in other pathogens, indicating that the method had good specificity.

### Sensitivity evaluation of the RPA-LFS-based GBS detection method in crude bacterial lysates, crude artificially contaminated urine, and purified artificially contaminated urine

**Figure 5:**
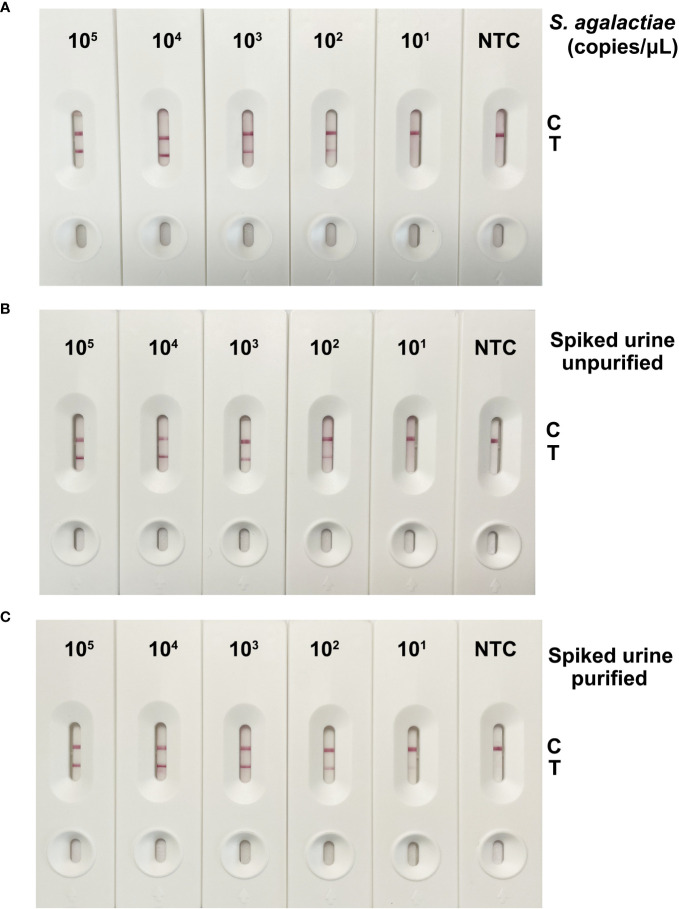
Results of detection sensitivity for the GBS detection method based on RPA-LFS. **(A)** The RPA-LFS results were obtained by using crude *S. agalactiae* solutions as the template, with final concentrations ranging from 10^5^ copies/μL to 10^1^ copies/μL. **(B)** The RPA-LFS results were determined by using crude *S. agalactiae* spiked urine as template with concentrations from 10^5^ copies/μL to 10^1^ copies/μL. **(C)** Detection results of RPA-LFS using genomic DNA purified from *S. agalactiae*-spiked urine, with final concentrations ranging from 10^5^ copies/μL to 10^1^ copies/μL as a template.

**Figure 5A** shows the results of RPA-LFS detection using *S. agalactiae* crude lysates as amplification templates, with concentrations ranging from 10^5^ copies/μL to 10^1^ copies/μL. With the decrease of template amount, the accumulation of cfb gene amplification products on the detection line gradually decreased. At a template amount of 10^2^ copies/μL, there were weak gold nanoparticles gathered at the T line. **Figures 5B, C** show the detection results of unpurified and purified *S. agalactiae* spiked urine samples, respectively, with a detection limit of 10^2^ copies/μL when using unpurified and an extremely weak detection limit of 10^1^ copies/μL when using purified urine samples. In conclusion, the detection sensitivity of the established on site GBS detection method is up to 10^2^ copies. The detection ability of low concentrations of GBS in urine may be reduced due to the degradation of genomic DNA by the urine matrix and the limitations of RPA’s tolerance for crude samples (Carrillo-Ávila et al., 2018).

### Coincidence rate analysis of GBS RPA-LFS and TaqMan Probe-based qPCR detection methods in recto-vaginal swabs

To evaluate the feasibility of the established GBS RPA-LFS detection method in clinical application, we simultaneously utilized the GBS RPA-LFS detection method and the TaqMan probe real-time quantitative PCR method to test 98 recto-vaginal swab genomic DNA samples. As shown in **Table 3**, the positive and negative detection rates for recto-vaginal swab samples using the RPA-LFS method were 12.24% (12/98) and 87.76% (86/98), respectively. Using the TaqMan probe real-time quantitative PCR method, the positive and negative detection rates for the samples were 8.16% (8/98) and 91.84% (90/98), respectively. The total coincidence rate between the two testing methods was 95.92%. Four positive differential samples (RPA positive and qPCR negative) were confirmed as GBS positive through bacterial culture, demonstrating that the detection method had high reliability.

**Table 3:** Clinical test results for recto-vaginal swabs.

Patients number	Detection results using different methods	
	RPA-LFS	TaqMan probe-based qPCR
356227	–	–
327876	–	–
484630	+	–
354802	–	–
1132429	–	–
661155	–	–
725443	–	–
292137	–	–
1122809	–	–
643241	–	–
567338	–	–
956016	–	–
768193	–	–
1129935	–	–
93586	–	–
1088126	–	–
320981	–	–
50476	–	–
886338	–	–
6451110	–	–
851065	–	–
1113662	–	–
1125717	–	–
1198779	–	–
1173276	–	–
288111	–	–
834332	–	–
367042	–	–
183664	–	–
1139072	–	–
158615	+	+
1176299	–	–
566199	–	–
389899	–	–
119598	–	–
1091837	–	–
518241	–	–
696280	–	–
1066668	–	–
263152	–	–
1094733	–	–
1118760	–	–
618223	–	–
409985	–	–
0001136460	+	+
292601	+	+
1137007	–	–
6001096363	+	+
0000474482	+	+
1135675	–	–
1105890	–	–
1131572	–	–
13900	–	–
1016742	–	–
1017415	–	–
1164781	–	–
378978	–	–
180968	–	–
1142377	–	–
1084844	–	–
881043	–	–
1135937	–	–
218097	–	–
391770	–	–
1102380	–	–
0061105430	+	+
0001048188	+	+
286258	–	–
1145247	–	–
1200534	+	+
1125065	–	–
337076	–	–
812119	–	–
761924	–	–
928520	–	–
986467	–	–
726647	–	–
1101798	–	–
429862	–	–
1113873	+	–
1771	–	–
582550	–	–
1194443	–	–
202045	–	–
794476	–	–
0001184280	–	–
83137	–	–
706	–	–
1007714	–	–
1113713	–	–
348684	–	–
324017	–	–
243801	+	–
38817	–	–
1136290	–	–
1108823	–	–
946550	+	–
948203	–	–

+ represents positive, - represents negative.

## Discussion

While GBS is not currently included in prenatal screening for pregnant women, its role in asymptomatic colonization and subsequent harm, such as perinatal infections leading to neonatal morbidity and mortality, is significant. (Armistead et al., 2019; Raabe and Shane, 2019). According to statistics, the mortality of premature infants infected with GBS is 30%, and early monitoring and intervention can substantially reduce neonatal deaths (Yuan et al., 2021). However, the widespread adoption of laboratory diagnosis for clinical GBS screening remains challenging, particularly in resource-limited areas (Yu et al., 2023). This study addresses this issue by employing RPA technology combined with nfo enzyme and LPS, enabling rapid, equipment-independent GBS detection suitable even in remote settings. This advancement in GBS detection will not only accelerate diagnosis but also expand access to prevention and control measures in underserved regions.

Based on the specificity of the capsular polysaccharides, GBS was categorized into nine serotypes: Ia, Ib, II, III, IV, V, VI, VII, and VIII. The serotypes Ia, Ib, II, III, and V were more frequently associated with the pathogenicity of GBS. Type III is the most virulent and infectious, causing more than half of serious infections. Type Ia was common in pregnant women; types Ia, Ib, II, and III were common in early neonatal infection; and type II and III were common in late neonatal infection (Russell et al., 2017; Dammann et al., 2022; Wadilo et al., 2023). Considering the multiple serotypes of GBS, the antigen–antibody test is prone to the risk of being false negative, leading this study to focus on the molecular diagnosis. RPA technology, with proven sensitivity and simplicity comparable with traditional PCR, has been applied diagnosing many diseases, including foot-and-mouth disease virus (Abd El Wahed et al., 2013), Dengue virus (Abd El Wahed et al., 2015), and *Mycobacterium tuberculosis* (Xu et al., 2021). While Yu et al. enhanced RPA’s detection sensitivity by combining it with cas12a and fluorescence detection (Yu et al., 2023), our study focuses on improving specificity and visualization. We used an improved RPA amplification reagent with an nfo enzyme and a specific probe with a THF site. This system allows for specifically cleave at the THF site, with visualization on LFS through the fluorescent labeling group on the probe and the biotin labeling on the primer, enhancing RPA’s utility in GBS detection. Although this method in GBS detection is novel, the innovation in system establishment is not original to this study.

GBS, an opportunistic pathogen, can transition from an asymptomatic mucosal carrier state to a severe invasive infection-causing agent. It adheres and colonizes human endothelial cells, penetrates the cell barrier, and evades host immunity through its surface molecules and secreted virulence factors, like the cfb hemolysin, causing host inflammatory storms and cell lysis (Herbert et al., 2004; Jin et al., 2018). We conducted homology analysis on the cfb gene of GBS strains from different sources and found that it has high conservation in different strains, indicating that this gene is a potential molecular diagnostic target for GBS. Studies by Gerolymatos et al. (2018); Lin et al. (2019), and Carrillo-Ávila et al. (2018) have validated the effectiveness of PCR and digital PCR methods based on the cfb gene for GBS detection in vaginal and rectal swabs, with high consistency with culture methods and correlation with clinical outcomes like premature membrane rupture and neonatal infections. These findings underpin the development of the GBS RPA detection method developed based on the cfb gene in this study, and the progression of GBS infection can be inferred by the intensity of gold nanoparticle aggregation on the detection line.

The healthy female vagina hosts a variety of pathogenic bacteria, with lactobacilli like *L. iners* and *L. crispatus* being predominant (Saraf et al., 2021). In addition, fungal *C. albicans* can cause candidal vulvovaginitis, and facultative anaerobic bacteria such as *L. monocytogenes*, which inhabit the vagina, cervix, etc., can also infect newborns through the placenta (Borges et al., 2011). Flagellates *T. vaginalis* causes trichomonal vaginitis (Kalia et al., 2020), and the presence of *E. cloacae* causes urinary tract infection (Akbari et al., 2016). Meanwhile, GBS is also a conditional pathogen that parasitizes the human lower digestive tract, and 10% to 30% of pregnant women also have this bacteria in the gastrointestinal tract. Therefore, we also selected several gastrointestinal pathogens to analyze the specificity of GBS. *Y. enterocolitica* is commonly found in feces, urine, and other causes of colitis (Ferreira et al., 2021). *K. pneumonia* is widely found in the respiratory tract and intestinal tract of the human body, causing pneumonia, enteritis, and so on (Lee and Kim, 2011). This study also selected our laboratory conserved *C. freundii*, *V. alginolyticus*, *V. parahaemolyticus*, *S. typhimurium*, *S. aureus*, and *P. aeruginosa* for specificity analysis of GBS screening. The results showed that the detection method established in this study could only specifically screen GBS, but not other common bacteria and pathogens in the vaginal, urinary, and intestinal tracts, thereby demonstrating its high specificity and reliability.

In evaluating clinical samples and analyzing the coincidence rate with TaqMan qPCR results, we used 98 unpurified recto-vaginal swab samples from the thermal lysis method and 98 purified recto-vaginal swab samples using a genomic DNA extraction and purification kit. In different templates, TaqMan qPCR detected 8 and 12 positive samples, with 90 and 86 negative samples, resulting in positive rates of 8.16% and 12.24%, respectively. RPA-LFS detected 12 positive samples in both cases, with 86 negative samples each, and the positive rate was 12.24% for both. These results suggest that TaqMan qPCR is more suitable for genomic DNA extraction and purification of samples in the laboratory and less suitable for amplifying crude DNA obtained by thermal lysis. The RPA-LFS system demonstrated high tolerance to sample purity, with no significant difference in detection results between the two methods. This aligns with previous reports on the RPA reaction’s ability to handle interference from complex templates (Dong et al., 2020). Even though we used the unpurified genomic DNA as a template to analyze the positive samples of the two methods, the coincidence rate was 95.92%, indicating that the GBS RPA-LFS detection platform established in this study has good consistency with the TaqMan qPCR detection method.

There are also some potential challenges in the application scenarios where RPA and LFS are combined. RPA technology is widely used because of its high sensitivity, but it also means that any small contamination can lead to a miscalculation of results. In addition, cross-contamination can be very dangerous when handling multiple samples, especially in a clinical setting. To solve these problems, we can adopt the following strategies. First, we should be physically isolated, using different physical spaces or equipment during the sample preparation, amplification, and detection phases to reduce the risk of cross-contamination. Secondly, we should carry out strict operating procedures and implement strict operating procedures, including the use of disposable tools, regular replacement of gloves, and the cleaning and disinfection of the workbench. Finally, in harsh environments, we can use nucleic acid scavengers to avoid cross-contamination within the same physical space.

## Conclusions

GBS infection should be detected and treated early. Therefore, it is of great significance to develop a universally applicable rapid detection technique for GBS. Considering the advantages of high accuracy, strong specificity, and high sensitivity of molecular biological methods, alongside the low equipment dependency of isothermal amplification and the visual readability of lateral flow chromatography test strips, this study combined RPA isothermal amplification technology, nfo enzyme cleavage THF site activity, and LFS visual analysis technology. We developed a highly reliable point-of-care testing method for rapid diagnosis based on the important GBS virulence factor, cfb hemolysin. This method can amplify GBS crude samples in 15 min at 39°C to 42°C and specifically detect GBS. Other pathogens, including *L. iners*, *L. crispatus*, *C. albicans*, *L. monocytogenes*, *Y. enterocolitica*, *K. Pneumoniae*, *E. cloacae*, *C. freundii*, *V. alginolyticus*, *V. parahaemolyticus*, *S. typhimurium*, *S. aureus*, *P. aeruginosa*, and *T. vaginalis*, showed no cross-reaction. The detection limit was 100 copies. The positive coincidence rate with TaqMan qPCR was 95.92% in 98 vaginal swabs. In conclusion, the rapid, sensitive, specific, visually interpretable, and device-independent GBS screening platform established in this study provides a feasible option for GBS screening in primary laboratories or at-home settings.

## Data availability statement

The original contributions presented in the study are included in the article/supplementary material. Further inquiries can be directed to the corresponding authors.

## Ethics statement

The studies involving humans were approved by Ethics Committee of Lianyungang Maternal and Child Health Hospital. The studies were conducted in accordance with the local legislation and institutional requirements. Written informed consent for participation in this study was provided by the participants’ legal guardians/next of kin.

## Author contributions

ML: Methodology, Writing – original draft. HW: Methodology, Writing – original draft. CC: Data curation, Writing – original draft. FM: Data curation, Writing – original draft. LS: Formal analysis, Investigation, Validation, Writing – original draft. TZ: Conceptualization, Project administration, Writing – review & editing. QM: Conceptualization, Project administration, Writing – review & editing.
